# Isothermal Cold Crystallization, Heat Resistance, and Tensile Performance of Polylactide/Thermoplastic Polyester Elastomer (PLA/TPEE) Blends: Effects of Annealing and Reactive Compatibilizer

**DOI:** 10.3390/polym8120417

**Published:** 2016-12-08

**Authors:** Sisi Wang, Sujuan Pang, Lisha Pan, Nai Xu, Tan Li

**Affiliations:** 1 College of Materials and Chemical Engineering, Hainan University, Haikou 570228, China; sisi.wang@ugent.be (S.W.); psjuan@hainu.edu.cn (S.P.); happylisap@hainu.edu.cn (L.P.); 2 Hainan Provincial Fine Chemical Engineering Research Center, Hainan University, Haikou 570228, China; 3 Shiner National & Local Joint Engineering & Research Center, Shiner Industrial Co., Ltd., Haikou 570125, China; tanlishiner@gmail.com

**Keywords:** poly(lactic acid), thermoplastic polyester elastomer, annealing, tensile property, heat resistance

## Abstract

The combined influences of crystallinity and reactive compatibilizer—a multifunctional epoxide (ADR)—on morphology, tensile performance, and heat resistance of polylactide/thermoplastic polyester elastomer (PLA/TPEE) (80/20) blends were investigated. Annealing involved an isothermal cold crystallization of PLA matrix was performed to increase crystallinity of the samples. First, isothermal cold crystallization kinetics were investigated using differential scanning calorimetry measurement. It was found that the addition of ADR decreased the crystallization rate of the samples. The maximum crystallinity of the annealed samples also decreased from 40% to 34% while ADR loading increased from zero to 1.0 phr. Furthermore, influence of crystallinity on mechanical performances of the PLA/TPEE sample was researched. The heat resistance of the sample showed a significant enhancement while increasing its crystallinity. Meanwhile, the tensile ductility of the crystallized PLA/TPEE sample became very poor due to the embrittlement with increased crystallinity and the incompatibility between PLA and TPEE. However, the annealed PLA/TPEE/ADR samples with high crystallinity kept a higher tensile ductility because ADR greatly improved the interfacial compatibility. Differences in tensile fracture behaviors of the quenched and annealed PLA/TPEE samples with and without ADR were discussed in detail. At last, crystallized PLA/TPEE/ADR blends with excellent heat resistance and high tensile ductility were obtained by annealing and reactive compatibilization.

## 1. Introduction

Due to their unique range of properties, polymeric materials currently have important roles in the lives and production of humanity. Innovation in polymers will thus make a valuable contribution to help solve environmental problems. In recent years, researchers have been paying increasing attention to renewable polymers due to the benefits of preserving the environment and saving petrochemical polymers. Due to its good biocompatibility, biodegradability, and high mechanical strength, poly(lactic acid) (PLA), produced from renewable resources (such as corn, sugarcane, beets, and so on) through bioconversion and polymerization, is being considered for use in a wide range of fields including electric appliance bodies, automobile components, and packaging materials, etc. [1,2,3,4]. However, its brittleness, slow crystallization rate, and poor heat resistance limit its usage in practical applications.

One of the promising approaches to enhance the toughness and ductility of PLA is blending it with other suitable polymers. Many elastomers and ductility polymers—such as polyamide 11 (PA 11), thermoplastic polyurethane (TPU), thermoplastic polyester elastomer (TPE), poly(butylenes-adipate-*co*-terephtalate) (PBAT), polycaprolactone (PCL), etc.—can be mixed into PLA matrix to increase the toughness and ductility of PLA materials [5,6,7,8,9].

As a block copolymer with hard segments of polybutylene terephthalate blocks and soft segments of poly(tetramethylene ether) glycol terephthalate blocks, thermoplastic polyester elastomers (TPEE) possess many excellent properties, such as strong thermal and mechanical properties. Due to its high toughness, TPEE is a good alternative for toughening PLA [6,7,10]. Maleic anhydride (MA)-grafted PLA (PLA-*g*-MA) was prepared to enhance the compatibility of PLA/TPE blends [6]. It was confirmed by IR spectroscopy that the anhydride groups of the PLA-*g*-MA chains reacted with the functional groups in the polybutylene terephthalate blocks of the TPE chains, and thus greatly enhanced the compatibilization between PLA matrix and TPE domains. SEM photographs indicated that the compatibilization effect of PLA-*g*-MA largely reduced the size of TPE domains. As a result, a higher level of surface area was produced for more interfacial interactions. Toughness results showed that Charpy impact toughness and fracture toughness (*K*_IC_ and *G*_IC_) of PLA increased greatly when the PLA/TPE samples were compatibilized with PLA-*g*-MA. For instance, *G*_IC_ of PLA increased as much as 166%. The toughening effect of TPEE on PLA was reported by Haydar U. Zaman et al. [7]. A diisocyanate compound (4,4′-Methylene *bis*(phenylisocyanate), MDI) was used as a reactive modifier to improve the compatibility of PLA and TPEE. With the incorporation of TPEE, the fracture mode of PLA transformed from classic brittle fracture to ductile fracture. Furthermore, the compatibilization effect of MDI led to a more remarkable increase (about 300%) in the tensile ductility of the PLA/TPEE sample, compared with that of the PLA/TPEE sample without MDI. The reactive compatibilization of MDI on the PLA/TPEE blends was attributed to the coupling reaction of PLA and TPEE macromolecules with MDI, because the isocyanate groups of MDI were highly reactive with the terminal –OH and –COOH groups of the polyesters. As a result, the interfacial adhesion and compatibilization between PLA matrix and TPEE domains were enhanced markedly. It was reported by our research team that a multifunctional epoxide (ADR) was used to enhance the compatibility and ductility of PLA/TPEE blend [10]. The epoxy groups of ADR chains reacted with carboxyl terminal and hydroxyl terminal of the polyesters, which extended the molecule chain and compatibilized the immiscible PLA and TPEE phases. With the incorporation of ADR, TPEE domains became smaller and phase interface became fuzzy, leading to a great improvement in the tensile ductility of the samples.

However, because of the slow crystallization rate of PLA matrix, PLA products prepared by melt processing moldings usually show a low crystallinity (below 10%), resulting in a poor heat resistance (Vicat softening temperature of around 60 °C) [11], which will restrict its applications in elevated temperature environments.

A couple of methods have been tested to improve the crystallinity and heat resistance of PLA materials, including annealing treatment [12], incorporation of nucleating agents [11,13], and a combination of annealing and nucleating agents [14], etc. It was found that annealing treatment and incorporation of a nucleating agent greatly improved the crystallinity of PLA, as well as enhancing its heat resistance. However, it was found that, with increasing the crystallinity, crystalline grains contacted each other and physical crosslinking points formed in the PLA matrix. Furthermore, the crystalline structure forced the random PLA chains and segments to arrange regularly and tightly, and free volume for macromolecule chains decreased, leading to a decrease of plastic deformation and an increase of brittleness of PLA matrix [15]. As a result, the increased crystallinity usually had a markedly negative effect on the ductility of PLA and its blends due to the brittleness and the vanishing of multiple crazes in the crack-tip region with crystallinity [16,17].

So far, the influences of crystallinity on microstructure, mechanical performance, fractured behavior, and heat resistance of PLA/elastomer blends are not well understood yet. How to prepare highly crystallized PLA blends with excellent ductility and heat resistance is still a challenging issue with huge practicability.

In the present study, two kinds of PLA blend—PLA/TPEE (80/20) and PLA/TPEE (80/20)—containing a small amount of reactive compatibilizer ADR, were prepared and subjected to annealing to obtain the highly crystallized samples. The main aim of the paper is to research the combined influences of crystallinity and reactive compatibilizer ADR on micro-morphology, tensile property, and heat resistance of the immiscible PLA/TPEE blends. The PLA/TPEE sample in glassy state was obtained by quenching, and annealing was performed to the quenched samples to obtain crystallized PLA/TPEE samples with high crystallinity. For the quenched PLA/TPEE sample in glassy state, annealing the sample above its glass transition temperature (around 60 °C) mainly involved the isothermal cold crystallization process of PLA in that the amorphous solid structure transformed into a highly crystallized solid structure [18]. Hence, in order to fully understand the effects of annealing and ADR on the crystallization behavior of PLA matrix, first, isothermal cold crystallization kinetics of PLA/TPEE blends with various ADR loadings were researched by using DSC measurement in the temperature range of 80–86 °C. Then, the combined effects of annealing and ADR loading on crystalline structure, tensile performance, heat resistance, and micro-morphology of the PLA/TPEE samples were studied by using differential scanning calorimetry (DSC), wide angle X-ray diffraction (WAXD), tensile test, vicat softening temperature (VST), and scanning electron microscope (SEM) observation, respectively. Finally, the differences in tensile fracture behaviors among the quenched and annealed PLA/TPEE samples with and without ADR were discussed.

## 2. Materials and Methods

### 2.1. Materials

PLA resin (4032D) was commercial available from Natureworks LLC (Minnetonka, MN, USA). Its density (*ρ*) and melting flow rate (MFR) were 1.25 g/cm^3^ and 5.3 g/10 min (190 °C under 2.16 kg load), respectively. TPEE resin (4056) was purchased from Dupont (Wilmington, DE, USA) (*ρ* = 1.17 g/cm^3^, and MFR = 6.0 g/10 min (190 °C under 2.16 kg load)). The multifunctional exipode oligomer (Joncryl ADR 4370s, abbreviating as ADR in this paper) purchased from BASF (Ludwigshafen, Germany) was used as reactive compatiblizer. Its molecule weight and exipode equivalent weight were 6800 and 285 g/mol, respectively.

### 2.2. Sample Preparation

Raw materials were placed in a vacuum oven at 50 °C for 12 h at least to remove moisture before each processing step. ADR was used as received.

PLA/TPEE blends with and without ADR were obtained by melt blending in an internal mixer (XS-60, Shanghai Kechuang Plastic Machinery Equipment Co., Ltd., Shanghai, China) at 190 °C with a rotor speed of 50 rpm for 10 min. The mass ratio of PLA/TPEE blend was set as 80:20 in weight fraction. The obtained PLA/TPEE/ADR ternary blends with different content of ADR (0.5 and 1.0 phr) were denoted as PLA/TPEE/ADR (80/20/0.5) and PLA/TPEE/ADR (80/20/1.0).

Quenched PLA/TPEE/ADR specimens for mechanical property measurements were prepared as follows: the obtained blends were molded under 210 °C with a pressure of 15 MPa within 8 min using a hot press, and then the melts in mold were quickly transferred into a cold press attached to a water cooling system and quickly cooled down to ambient temperature with a cooling rate of around 100 °C/min under 15 MPa. Finally, quenched sheets with 1 mm thickness were obtained. The dumbbell shaped specimens were cut from the sheets for the following tests.

Crystalline PLA specimens were prepared as follows: a part of the quenched specimens were annealed in an oven for different time (holding time ranged from 0 to 60 min) under 80 °C to obtain the crystallized specimens with varying crystallinity. In the paper, it has to be noted that the specimens with a holding time of 0 min refer specifically to the quenched (as-molded) specimens without annealing.

The samples were kept at room temperature for 72 h prior to testing.

### 2.3. Differential Scanning Calorimetry (DSC) Analysis

Crystallization behavior of the samples was investigated using a TA Instruments Q100 series (TA Instruments, New Castle, DE, USA) under nitrogen atmosphere with a flow rate of 50 mL/min.

In order to research the isothermal cold crystallization behavior of the samples, each sample (around 5 mg) was first heated quickly to 200 °C and held at 200 °C for 4 min to erase its thermal history; then quenched down to 30 °C at a rate of 50 °C/min; and finally, heated again quickly to a preset crystallization temperature (*T*_c_ = 80, 82, 84 and 86 °C, respectively) at 200 °C/min, and kept at the set temperature for enough time to crystallize completely. The exotherm of each sample was recorded.

The isothermal crystallization kinetics parameters of polymers can be obtained by the well-known Avrami equation, as shown in Equation (1).

*X*(t) = 1 − exp(−*kt^n^*), (1)

where *t* denotes the real time of crystallization. *X*(t) is related to the relative crystallinity at time *t*. *k* stands for the Avrami rate constant, and *n* is the Avrami exponent depending on the nucleation mechanism and crystal growth pattern.

According to the obtained isothermal DSC thermograms, *X*(t) is the ratio of the area of the exotherm up to time *t* (*Q_t_*) divided by the area of the total exotherm (*Q*_∞_):

(2) $$X\left(t\right)=\frac{Q_{t}}{Q_{\infty }}=\frac{\int_{0}^{t}\left(dH/dt\right)dt}{\int_{0}^{t}\left(dH/dt\right)dt}$$

where *Q_t_* is the heat generated at time *t*, and *Q*_∞_ is the heat generated at infinite time. *dH*/*dt* refers to the rate of heat evolution.

Taking the Avrami equation double logarithmic form, Equation (2) can be transformed to Equation (3).

log[−ln(1 − *X*(t))] = *n*log*t* + log*k*, (3)

The crystallization half-time (*t*_1/2_), defined as the time required to reach half of the final crystallinity, can be calculated according to Equation (4).

*t*_1/2_ = (ln2/*k*)^1/*n*^, (4)

Meanwhile, the crystallinities of the quenched and annealed specimens were obtained by using DSC measurement, the testing conditions were as follows: each sample was heated directly to 200 °C at a scan rate of 10 °C/min from 30 °C. Cold crystallization enthalpy (∆*H*_cc_) and fusion enthalpy (∆*H*_m_) shown in the heating scan were recorded. The crystallinity (*X*_c_) of each specimen was calculated by Equation (5).

(5) $$X_{c}=\frac{\left|\Delta H_{\mathrm{cc}}+\Delta H_{m}\right|}{\Phi \times \left|\Delta H_{m}^{*}\right|}$$

where ∆*H*_cc_ and ∆*H*_m_ are cold crystallization enthalpy and melting enthalpy of the PLA samples, respectively; Φ stands for the mass fraction of PLA and $\Delta H_{m}^{*}$ refers to the melting enthalpy of 100% crystalline PLA, which is reported to be 93 J/g [19].

### 2.4. Wide Angle X-Ray Diffraction (WAXD)

Wide angle X-ray diffraction (Bruker D8 ADVANCE, Karlsruhe, Germany) was employed to evaluate the crystalline structure of the quenched and annealed samples in the reflection mode. The CuKα radiation (λ = 1.54 Å) was operated at 40 kV and 40 mA. Data were recorded in the range of 5° ≤ 2θ ≤ 50° with a scan speed of 2°/min.

### 2.5. Vicat Softening Temperature (VST) Analysis

The VST of the quenched and annealed samples were investigated using a thermal deformation and VST tester (Chengde Jiande Detection Instrument Co., Ltd., Chengde, China). The test was conducted according to Standard GB/T 1633–2000 with a heating rate of 120 °C/h and a load of 1000 g.

For each formulation, pairs of specimens were tested until the VST values of the two specimens did not differ by more than 2 °C. The average value of the VST was then calculated.

### 2.6. Tensile Tests

The tensile tests were done by using an intelligent electronic tensile testing instrument (XLW, Jinan Lan-Guang Mechanical and Electrical Technology Co., Ltd., Jinan, China) with a crosshead speed of 50 mm/min and a gauge length of 30 mm. The samples were stored at rest for 72 h at room temperature before testing. Each value is the average of five valid tests.

### 2.7. Micro-Morphology of the Samples

The micro-morphology of the samples was examined using scanning electron microscope (SEM) (S-3500N, Hitachi, Tokyo, Japan). The samples were immersed in liquid nitrogen for enough time and then broke quickly to obtain the cryo-fractured surfaces. The cryo-fractured surfaces of the samples were sputtered with gold powder in order to achieve better SEM observations. An acceleration voltage of 10 KV was used for the SEM examination. The magnification was 3000 times.

## 3. Results and Discussion

### 3.1. Isothermal Cold Crystallization Kinetics

**Figure 1:**
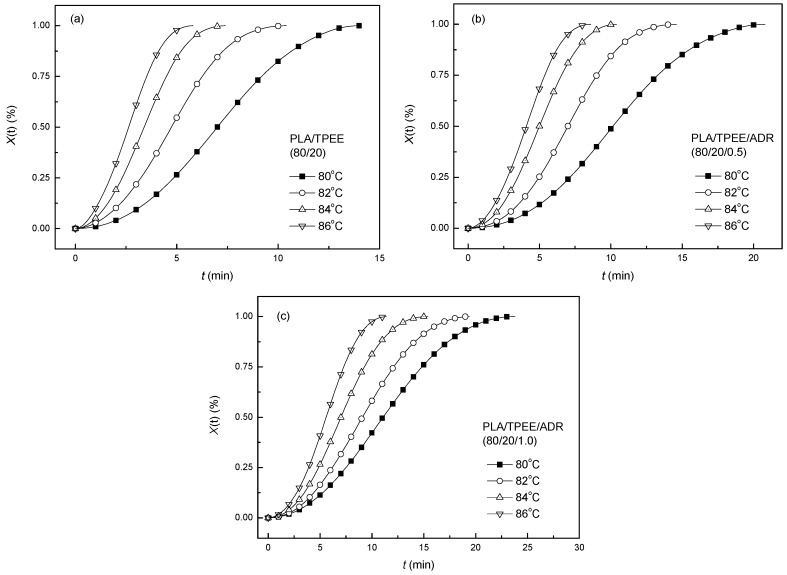
Relative crystallinity (*X*(t)) vs. crystallization time (*t*) in the isothermal cold crystallization process for PLA blends: (**a**) PLA/TPEE (80/20); (**b**) PLA/TPEE/ADR (80/20/0.5); (**c**) PLA/TPEE/ADR (80/20/1.0).

Isothermal cold crystallization behaviors of the PLA/TPEE (80/20) samples with and without ADR were investigated by DSC measurement. Figure 1 showed the relationship between relative degree of crystallinity (*X*(t)) and isothermal time (*t*) of the samples at different crystallization temperature (*T*_c_ = 80, 82, 84 and 86 °C, respectively). All the curves showed a sigmoid dependence on isothermal time. The sigmoid curves moved to the left with increasing *T*_c_, indicating that time to reach the maximum crystallinity for PLA blends decreased under higher *T*_c_. The dependences of crystallization half-time (*t*_1/2_) on *T*_c_ and ADR loading had been further discussed in succession by calculating from isothermal crystallization kinetics of the PLA/TPEE blends.

**Figure 2:**
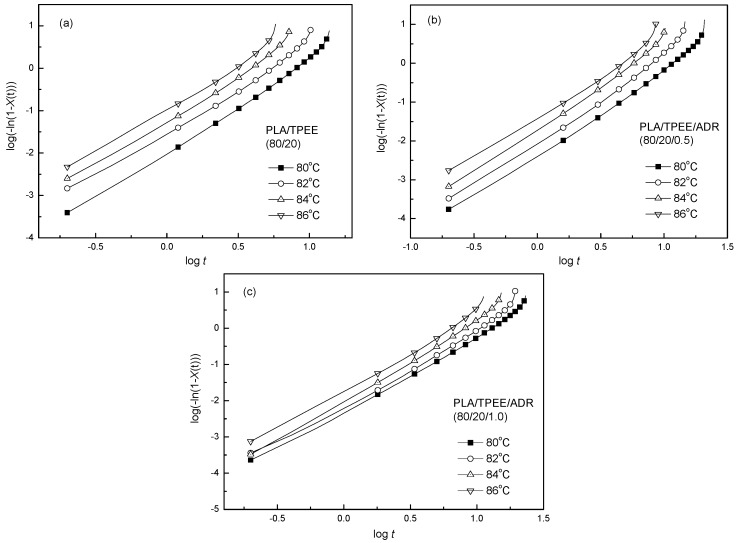
Plots of log[−ln(1 − *X*(t))] vs. log*t* of the representative samples isothermally crystallized at the predetermined temperatures: (**a**) PLA/TPEE (80/20); (**b**) PLA/TPEE/ADR (80/20/0.5); (**c**) PLA/TPEE/ADR (80/20/1.0).

The representative plots of log[−ln(1 − *X*(t))] versus log*t* for the PLA/TPEE blends were shown in Figure 2. The curves consisted of two parts: the first crystallization stage and the second crystallization stage. It was found that in the first stage log*t* linearly depended on log[−ln(1 − *X*(t))]. At the second stage, an obvious roll-off at longer times was observed, indicating that the existence of a secondary crystallization which resulted from the spherulite impingement in the later stage of the crystallization process at a longer crystallization time. Hence, the linear regimes were chosen to determine the exponent *n* and *k* of the samples according to Equation (3). The calculated crystallization kinetics parameters were listed in Table 1.

Usually, the value of *n* should be an integer between 1 and 4 for different nucleation mechanisms and crystal growth modes. As shown in Table 1, the values of *n* were in the range of 2.2–2.5 for the PLA/TPEE sample at crystallization temperatures ranged from 80 to 86 °C. It was an average value of various nucleation mechanisms, and growth dimensions occurred simultaneously in the crystallization process. As a result, the Avrami exponent *n* was not a straightforward integer.

Due to the existence of TPEE domains, it can be inferred that the nucleation type of PLA in the blends should predominantly be heterogeneous nucleation and its growth dimension should be 2D growth. By contrast, the values of *n* were also kept between 2.3 and 2.5 for the PLA/TPEE/ADR samples at the same crystallization temperatures. It meant that the incorporation of ADR has little effect on the crystallization mechanism of PLA matrix.

The crystallization half-time (*t*_1/2_) and crystallization induction time (*t*_induce_) [20] of the PLA/TPEE and PLA/TPEE/ADR samples at different crystallization temperature (*T*_c_ = 80, 82, 84 and 86 °C) were also listed in Table 1. It was shown that *t*_induce_ and *t*_1/2_ of the PLA/TPEE (80/20) sample was 8.7 and 6.9 min under 80 °C, respectively. As the cold crystallization temperature increased to 86 °C, *t*_induce_ and *t*_1/2_ of the PLA/TPEE (80/20) sample shortened to 1.9 and 2.6 min, respectively. It was attributed to the fact that the elevated temperature was helpful for enhancing the macromolecule chain mobility and arrangement ability, leading to an improvement in cold crystallization rate of PLA matrix.

Furthermore, compared to the PLA/TPEE (80/20) sample, *t*_induce_ and *t*_1/2_ of the PLA/TPEE/ADR (80/20/*x*) samples tended to prolong at the same crystallization temperature. For example, when *T*_c_ was 80 °C, *t*_induce_ and *t*_1/2_ increased from 8.7 and 6.9 min for the PLA/TPEE (80/20) sample up to 11.5 and 11.2 min for the PLA/TPEE/ADR (80/20/1.0) sample. It indicated that the incorporation of ADR impeded the cold crystallization ability of PLA matrix. The depression of crystallization ability can be ascribed to the restraining in PLA segment reorganization in the presence of ADR. Multiple epoxide groups on ADR reacted with terminal –COOH groups and terminal –OH groups on PLA and TPEE chains leading to the formation of extended and branched chains [10]. The chain extension/branching results are complex. Besides the formation of PLA-ADR-TPEE copolymers, the generation of PLA-ADR-PLA and TPEE-ADR-TPEE is inevitable when ADR is melt blended with PLA/TPEE blend. The SEC measurement’s result showed that the molecule weights of PLA and TPEE increased largely with the incorporation of a small amount of ADR [10]. The increase in molecule weight, as well as the formation of long branched chains, would enhance macromolecular chain entanglement and significantly limit the regular arrangement ability of PLA segments and chains in amorphous solid, especially when the cold crystallization process was held in a relatively lower temperature (close to *T*_g_). Thereby, the crystal growth of PLA matrix was retarded in this situation. As a result, the overall crystallization rate of the samples showed a decrease with the incorporation of ADR at crystallization temperatures ranging from 80 to 86 °C.

It was known from the crystallization kinetics analysis of the PLA/TPEE/ADR blends that the isothermal cold crystallization ability of the PLA/TPEE samples was retarded to some extent when ADR increased from 0 to 1.0 phr. However, the semi-crystallized PLA/TPEE/ADR samples still could be obtained by appropriately prolonging the crystallization time. In the subsequent experiments, the quenched samples were annealed at 80 °C in order to obtain the crystallized samples. The combined effects of annealing and ADR loading on crystalline structure, tensile performance, heat resistance, and micro-morphology of the PLA/TPEE samples have been researched and discussed.

### 3.2. Effect of Annealed Time on Crystallinity of PLA/TPEE/ADR Samples

**Figure 3:**
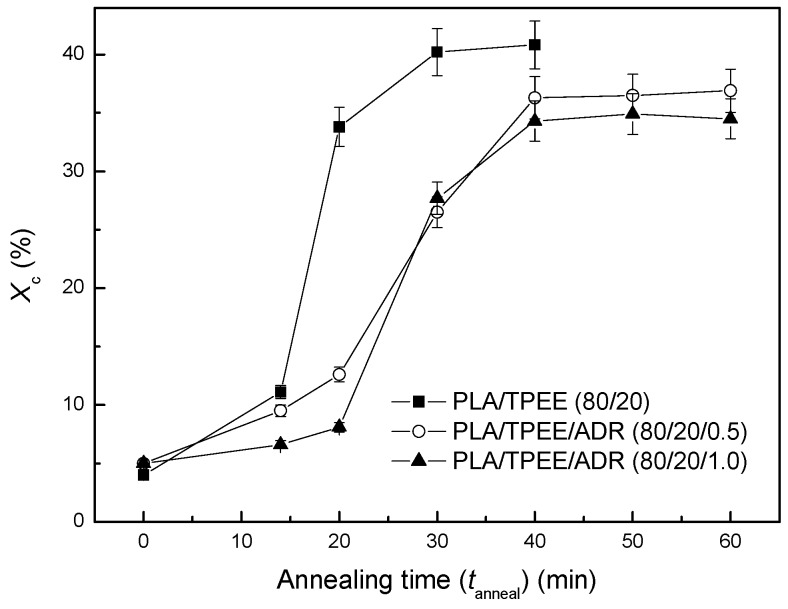
Crystallinity (*X*_c_) vs. annealed time of PLA/TPEE/ADR (80/20/*x*) samples annealed at 80 °C.

Plots of annealed time vs. crystallinity (*X*_c_) of the PLA/TPEE/ADR (80/20/*x*) samples annealed at 80 °C are shown in Figure 3. It was found that crystallinity of the samples increased markedly while increasing the annealed time due to the cold crystallization of PLA matrix during the annealing process. It was consistent with the isothermal cold crystallization kinetics results. As seen in Figure 3, the annealed times to reach the maximum crystallinity for the PLA/TPEE (80/20), PLA/TPEE/ADR (80/20/0.5) and PLA/TPEE/ADR (80/20/1.0) samples were 30, 40 and 40 min, respectively. Then, the crystallinities of the samples kept almost constant. As we know, the annealed time to reach the maximum crystallinity should consist of induced time and crystallization time. With the incorporation of ADR, the annealed time to reach the maximum crystallinity showed an obvious increase due to the fact that the chain extension and branched reactions among PLA, TPEE, and ADR impaired the cold crystallization ability of the PLA matrix.

Moreover, the maximum crystallinity of the PLA/TPEE samples also decreased with the increasing of ADR loading. As shown in Figure 3, while the annealed time reached to 30 min, *X*_c_ of the PLA/TPEE (80/20) sample increased from 4% for the quenched PLA/TPEE sample to the maximum value of 40%. A similar result was also reported by Harris et al. [14]. It was confirmed that annealing of both nucleated and neat PLA samples at 80 °C increased the crystallinity of PLA to its maximum level of around 42%. However, with increasing ADR loading, the maximum *X*_c_ of the PLA/TPEE/ADR (80/20/0.5) and PLA/TPEE/ADR (80/20/1.0) samples dropped down to 36% and 34%, respectively. The reduction in the maximum crystallinity could also be explained by the fact that the extended chain and branched reactions among PLA/PLA/ADR and PLA/TPEE/ADR reduced the regularity of PLA chains and weakened their regular arrangement ability.

**Figure 4:**
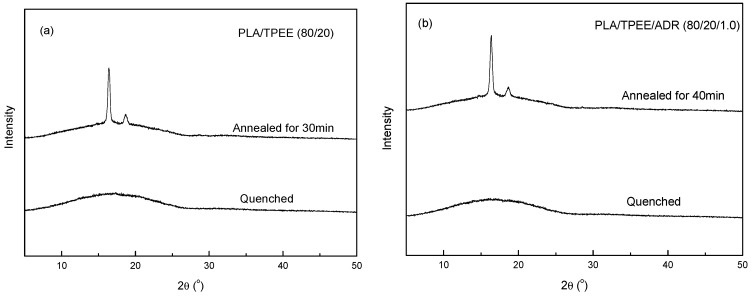
WAXD patterns of the quenched samples and the annealed samples held at temperature of 80 °C, (**a**) PLA/TPEE (80/20) samples; (**b**) PLA/TPEE/ADR (80/20/1.0) samples.

Meanwhile, WAXD measurement was conducted to investigate crystal structures of the quenched and annealed PLA/TPEE/ADR blends. WAXD patterns of the quenched and annealed PLA/TPEE/ADR (80/20/1.0) samples were presented in Figure 4. As a comparison, the WAXD patterns of the quenched and annealed PLA/TPEE (80/20) samples were also presented in Figure 4. In order to obtain the fully annealed samples, the quenched PLA/TPEE (80/20) and PLA/TPEE/ADR (80/20/1.0) samples had been held at 80 °C for 30 and 40 min, respectively. As seen in Figure 4, the quenched PLA/TPEE/ADR (80/20/1.0) sample, as well as the quenched PLA/TPEE (80/20) sample showed a broad dispersing diffraction peak, indicating that the samples were almost amorphous with very low crystallinity. However, two strong reflections located at 16.4° and 18.7° were observed in the WAXD pattern of the PLA/TPEE/ADR (80/20/1.0) sample annealed at 80 °C for 40 min, as well as in that of the PLA/TPEE (80/20) sample annealed at 80 °C for 30 min. It was confirmed that crystal modification of the annealed PLA/TPEE and PLA/TPEE/ADR samples could be assigned to disorder α form of PLA, named α′ form. By using WAXD, DSC, and infrared spectroscopy (IR) measurements, Zhang et al. found that, dependent on *T*_c_, the disorder α′ and order α modification of PLA were formed at a low temperature range (*T*_c_ < 100 °C) and high temperature range (*T*_c_ > 120 °C), respectively [21,22]. It was found that the two strong diffraction peaks between the two samples showed distinct shifts in the 2θ positions. In the case of PLA sample annealed at 140 °C, the two strong reflections located at 16.7° and 19.1°, which were characteristic reflections of α modification. However, the two reflections were found at 16.4° and 18.7° in WAXD pattern of the PLA sample annealed at 80 °C, which were attributed to α′ modification. In addition, it was confirmed by the authors that a mixture of α and α′ phases had formed in PLA matrix while the samples crystallized in the temperature range from 100 to 120 °C. It revealed from Figure 4 that the isothermal cold crystallization of the quenched PLA/TPEE samples with and without ADR happened while the samples were annealed at 80 °C. After being fully annealed, the highly crystallized PLA/TPEE and PLA/TPEE/ADR blends with α′ phases were obtained.

### 3.3. Effects of ADR and Crystallinity on Tensile Performance, Heat Resistance, and Micro-Morphology of the PLA/TPEE Samples

**Figure 5:**
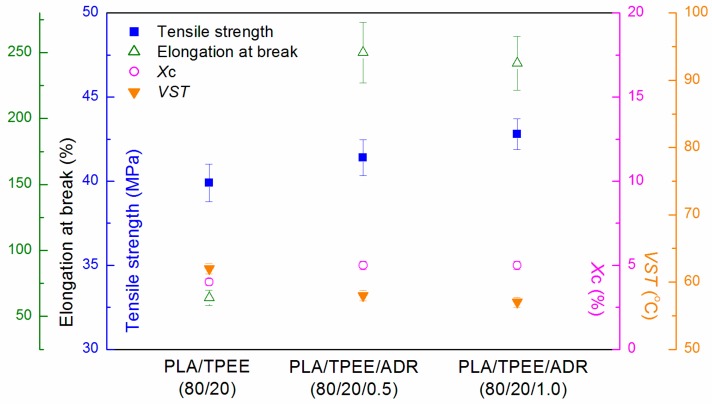
Data of tensile performances, *X*_c_ and VST of quenched PLA/TPEE/ADR (80/20/*x*) blends.

Tensile performance, crystallinity, and VST of the quenched PLA/TPEE samples were shown in Figure 5.

As Figure 5 showed, the tensile ductility of the quenched PLA/TPEE samples improved greatly with the incorporation of ADR. Compared to the quenched PLA/TPEE (80/20) sample without ADR, the elongation at break of the quenched PLA/TPEE/ADR (80/20/0.5) sample reached 250%, having increased by 290% (64% for the quenched PLA/TPEE). While ADR loading further increased up to 1.0 phr, the elongation at break kept steady. Meanwhile, the tensile strength of the PLA/TPEE samples increased from 39.9 to 42.8 MPa with increasing ADR addition from 0 up to 1.0 phr, because ADR as a chain extender increased the molecular weight of PLA and therefore increased the strength with longer chains [23]. With longer chains, there were more entanglements and interactions among these polymer chains, making them relatively more stationary. This, in turn, increased the strength of the PLA samples.

**Figure 6:**
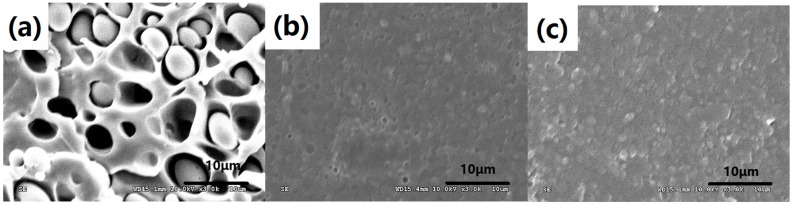
SEM micrographs of quenched PLA/TPEE specimens with and without ADR: (**a**) PLA/TPEE (80/20); (**b**) PLA/TPEE/ADR (80/20/0.5); (**c**) PLA/TPEE/ADR (80/20/1.0).

As is reported in our previous research [10], the in situ compatibilization mechanism of the PLA/TPEE/ADR blends was studied systematically. When ADR was added during the melt blending process, the terminal –COOH group and terminal –OH group of PLA and TPEE reacted with the multiple epoxides on ADR. ADR not only played the role of chain extender, but also played the role of reactive compatibilizer by forming PLA-ADR-TPEE copolymer, which reduced the interface energy, improved the interface adhesion, and thus decreased the dispersed domain size. As a result, tensile ductility of the blends increased significantly. Similarly, Racha Al-Itry et al. studied the impact of ADR on the interfacial property of PLA/PBAT blends [8]. The interfacial tension in the modified/compatibilized PLA/PBAT decreased, and formed the PLA-ADR-PBAT copolymer. The improvement in mechanical properties also was attributed to the formation of the PLA-ADR-PBAT copolymer. Hence, it was supposed that the coupling reaction between ADR and PLA/TPEE improved the compatibility of the blends. The compatibilization effect of ADR on PLA/TPEE samples can be verified by SEM micrographs presented in Figure 6. As shown in Figure 6a, it was found that the interface compatibility of TPEE domains and PLA matrix was poor according to the fact that the sizes of TPPE particles were larger (around 5 μm) and marked debonded interfaces existing in the PLA/TPEE (80/20) sample. However, for the PLA/TPEE/ADR (80/20/0.5) and (80/20/1.0) samples (Figure 6b,c), due to the reactive compatibility of ADR, the TPEE domain sizes markedly dropped down to 1 μm or less from 5 μm, and the phase interface became much fuzzier.

Although the addition of ADR improved the tensile performance of the PLA/TPEE blends obviously, the slow melt-crystallization rate of PLA led to low crystallinity (*X*_c_) for the quenched PLA/TPEE samples. As shown in Figure 5, the crystallinities of the quenched PLA/TPEE samples with and without ADR were only around 5%, indicating that the PLA samples were almost amorphous. The WAXD patterns presented in Figure 4 also confirmed the full glassy state in the quenched PLA/TPEE samples. As we knew, the glassy state of PLA matrix would lead to a poor heat resistance of the quenched PLA/TPEE samples, because of the low glass transition temperature (around 60 °C) of PLA.

As expected, it was found from Figure 5 that the VST value of each quenched PLA/TPEE samples with and without ADR was very low (about 60 °C). The poor heat resistance would restrict applications of the PLA/TPEE blends with low crystallinity in elevated temperature environments.

It can be seen in Figure 3 that *X*_c_ of the PLA/TPEE samples with and without ADR increased significantly with the increase of annealing time at 80 °C. While the annealing time reached to 30 min, *X*_c_ of the PLA/TPEE (80/20) sample increased from 4% for the quenched PLA/TPEE sample up to a maximum value of 40%. Meanwhile, while the annealing time lengthened to 40 min, *X*_c_s of the PLA/TPEE/ADR (80/20/0.5) and PLA/TPEE/ADR (80/20/1.0) samples also reached to maximum values of 36% and 34%, respectively. Apparently, *X*_c_ of the quenched PLA/TPEE samples could be markedly increased by annealing treatment. As a result, the highly crystallized PLA/TPEE samples could be obtained after fully annealed at 80 °C.

**Figure 7:**
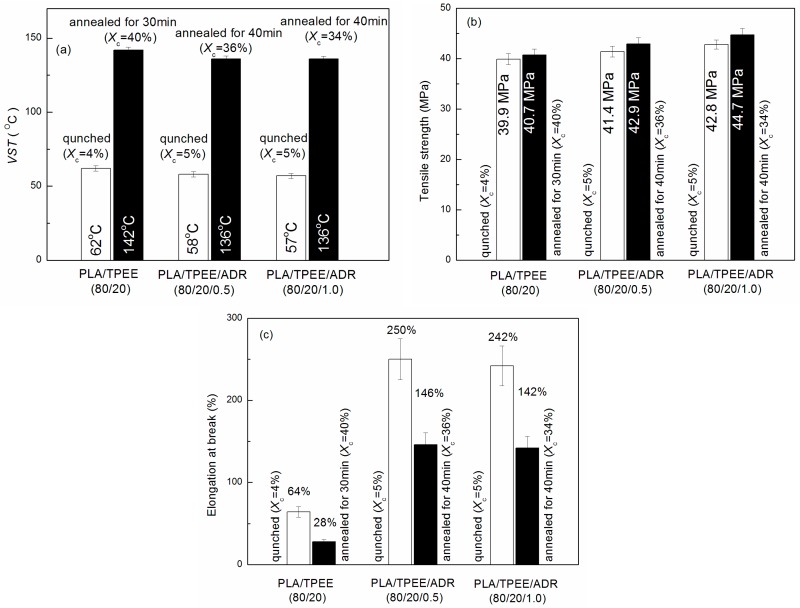
Effects of annealing and ADR loading on VST, tensile strength, and elongation at break of PLA/TPEE/ADR (80/20/*x*) samples: (**a**) VST; (**b**) tensile strength; and (**c**) elongation at break.

Figure 7 presented the influences of annealing and ADR loading on crystallinity, VST, tensile strength, and elongation at break of the PLA/TPEE/ADR (80/20/*x*) samples.

As Figure 7a showed, when the quenched PLA/TPEE (80/20) had been annealed at 80 °C for 30 min, its *X*_c_ increased from 4% to 40%. The VST of the annealed PLA/TPEE sample increased greatly from 62 to 142 °C. The PLA/TPEE/ADR (80/20/0.5) and (80/20/1.0) samples also showed a similar *X*_c_ dependence. For example, after annealing at 80 °C for 40 min, *X*_c_ of the PLA/TPEE/ADR (80/20/1.0) sample increased from 5% to 34%, and its VST value increased significantly from 57 to 136 °C.

The improvement in VST with increasing *X*_c_ could be explained by the transformation of the amorphous phase into crystal phases in the PLA matrix during the cold crystallization caused by the annealing treatment. For the quenched PLA/TPEE samples, the crystallinity was only around 5%. It meant that the PLA matrix (continuous phase) was almost amorphous. As a result, the heat resistances of the PLA/TPEE samples were poor, and their VSTs were dominated mostly by *T*_g_ of PLA matrix. However, when the quenched PLA/TPEE samples were annealed at 80 °C, a cold crystallization process of PLA matrix happened. An amount of crystal nuclei formed and constantly grew to form crystalline structures from the amorphous PLA matrix. With the lengthening of annealing time, the crystals continued to grow in PLA matrix until these crystals fully developed and impinged upon each other. When *X*_c_ of the PLA/TPEE sample reached to the maximum, the fully grown crystals squeezed each other, which led to the formation of physical crosslinking in the PLA matrix. Meanwhile, the cold crystallization of the PLA matrix was terminated almost due to the huge steric hindrance resulted from the physical crosslinking. Thus, the significant enhancement in the heat resistance of the PLA/TPEE samples can be attributed to the restraint of molecular motion by the formation of firm crystals and physical crosslinking [11,17]. The highly crystallized PLA/TPEE samples did not show a significant thermal deformation until the environment temperature increased close to *T*_m_ of crystalline PLA. In the elevated temperature range, the physical crosslinking points were gradually destroyed and the crystallized PLA started to fuse, that made the PLA/TPEE samples markedly deform under the external loading. In that case, the VST increased markedly because it was dominated by *T*_m_ (around 168 °C) of crystalline PLA matrix, instead of *T*_g_ of amorphous PLA phase [11].

In Figure 7a, all the PLA/TPEE (80/20) samples with and without ADR showed a similar *X*_c_ dependence of VST. However, it was found that the maximum value of VST for the annealed PLA/TPEE/ADR (80/20/0.5) and (80/20/1.0) samples decreased from the maximum VST of 142 °C for the PLA/TPEE (80/20) sample to 136 °C. It was due to the reduction in the maximum *X*_c_ of the PLA/TPEE/ADR samples (Figure 3) when ADR was incorporated as a chain extender that weakened the cold crystallization ability of PLA chains.

In summary, the increase of crystallinity due to annealing greatly enhanced the heat resistances of the PLA/TPEE samples with and without ADR. However, it is well known that the formation of firm crystals and physical crosslinking in polymer matrix would markedly restrain the molecular motion resulting in embrittlement of the semi-crystalline polymer [24]. Hence, it is necessary to research the influence of *X*_c_ on the tensile ductility and strength of the PLA/TPEE samples with and without ADR.

**Figure 8:**
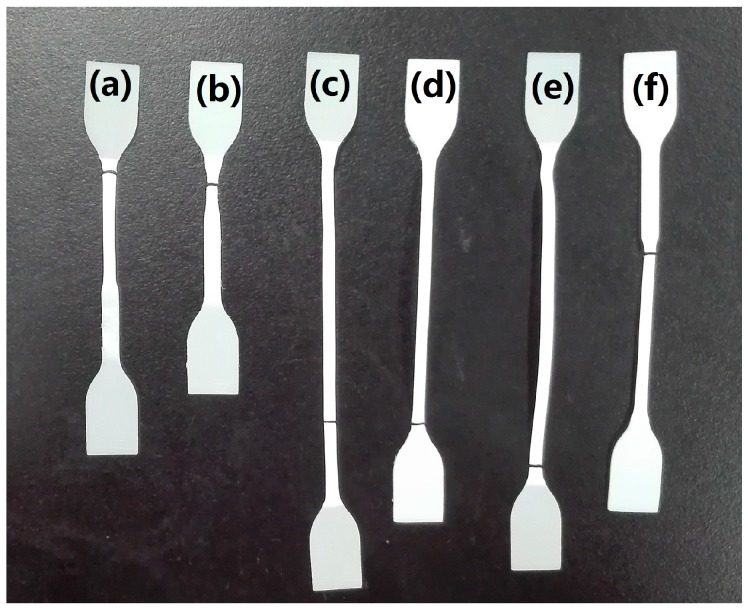
Optical photograph of the tensile-fractured specimens: (**a**) Quenched PLA/TPEE (80/20) specimen with *X*_c_ of 4%; (**b**) Annealed PLA/TPEE (80/20) specimen with *X*_c_ of 40%; (**c**) Quenched PLA/TPEE/ADR (80/20/0.5) specimen with *X*_c_ of 5%; (**d**) Annealed PLA/TPEE/ADR (80/20/0.5) specimen with *X*_c_ of 36%; (**e**) Quenched PLA/TPEE/ADR (80/20/1.0) specimen with *X*_c_ of 5%; (**f**) Annealed PLA/TPEE/ADR (80/20/1.0) specimen with *X*_c_ of 34%.

The effects of annealing and ADR loading on tensile strength and elongation at break of the PLA/TPEE/ADR (80/20/*x*) samples were presented in Figure 7b,c, respectively. In addition, a photograph of the tensile-fractured PLA/TPEE (80/20) and PLA/TPEE/ADR(80/20/*x*) specimens is shown in Figure 8.

As seen in Figure 7b, the tensile strength of the PLA/TPEE (80/20) samples showed a slight increase with the increase of *X*_c_. For the PLA/TPEE (80/20) samples with ADR, a similar increasing trend of tensile strength to the PLA/TPEE (80/20) samples was also observed. For example, while increasing *X*_c_ of the PLA/TPEE/ADR (80/20/1.0) sample from 5% to 34%, the tensile strength increased from 42.8 to 44.7 MPa. The moderate enhancement in tensile strength of the PLA/TPEE samples was attributed to the formation of firm crystals and physical crosslinking in the samples with the increase of *X*_c_. Moreover, it was interesting to note that the tensile strength also increased upward slightly while ADR loading increased from 0 to 1.0 phr. It was attributed to the increased molecular weight caused by the chain extension reaction between ADR and PLA chains [10]. Hence, the *X*_c_ and ADR showed a synergistic enhancement on the tensile strength of the PLA/TPEE samples.

Although the increase of *X*_c_ enhanced the heat resistance and tensile strength of the PLA/TPEE (80/20) samples with and without ADR, it was found from Figure 7c that the tensile ductility of the samples showed a significant decrease. For the PLA/TPEE (80/20) sample, the elongation at break decreased drastically from 64% to 28% while the *X*_c_ increased from 4% to 40%. Compared with that of the PLA/TPEE (80/20) sample, the elongation at break of the PLA/TPEE/ADR (80/20/0.5) sample decreased from 250% to 146% while its *X*_c_ increased from 5% to 36%. However, when the ADR loading increased to 1.0 phr, the elongation at break of the annealed sample remained almost unchanged. It was apparent that the significant increase of *X*_c_ markedly reduced the tensile ductility of each PLA/TPEE sample. It was due to the fact that the progress of crystallization and increase of *X*_c_ in the semi-crystalline PLA samples resulted in an increased embrittlement of the samples and hence decreased the tensile ductility. As shown in Figure 8, necking and whitening phenomena occurred simultaneously while the PLA/TPEE samples with and without ADR were cold drawn during the tensile test. Therefore, the tensile deformation mechanism of the PLA/TPEE samples should be attributed to the crazing with shear yielding mechanism. In general, the generation, development, and termination of crazes plays an important role in the toughening of polymer materials. Furthermore, for polymer blends, the interfacial compatibility and dispersed phase sizes have a great effect on the initiation and termination of crazes that will directly affect the tensile ductility of the blends.

**Figure 9:**
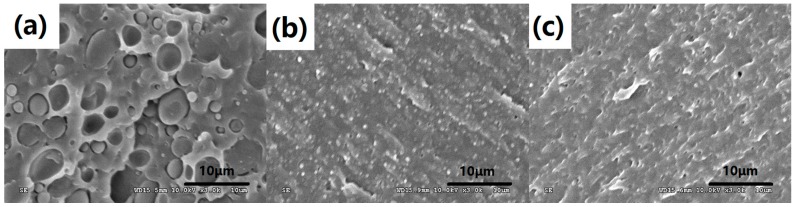
SEM micrographs of PLA/TPEE/ADR samples annealed at 80 °C for 40 min: (**a**) PLA/TPEE (80/20); (**b**) PLA/TPEE/ADR (80/20/0.5); (**c**) PLA/TPEE/ADR (80/20/1.0).

Figure 9 presented SEM micrographs of the annealed PLA/TPEE (80/20) samples with and without ADR which had been annealed at 80 °C for 40 min, respectively. The PLA/TPEE (80/20) samples annealed for 40 min showed marked interface debondings due to the poor adhesion between PLA matrix and TPEE domains, as shown in Figure 9a. The poor adhesion and interface debonding phenomenon was also observed in the quenched PLA/TPEE (80/20) sample (Figure 6a). It is well-known that the weaker interfacial regions could not effectively terminate the growth of crazes. As a result, the cracks would be induced and rapidly develop through the weaker interfacial regions, leading to a poor tensile ductility and low elongation at break for the quenched PLA/TPEE (80/20) sample during the tensile test. While the annealed time increased to 40 min, the crystallinity of the PLA/TPEE (80/20) sample increased from 4% for the quenched sample to the maximum value of 40%. It was confirmed that a stacking effect of the increased crystallinity and poor compatibility of PLA and TPEE markedly weakened the tensile ductility of the annealed PLA/TPEE (80/20) sample with high crystallinity. Sang Dae Park et al. researched the influences of *X*_c_ and loading-rate on the fracture behavior of PLA [16,17]. It was found that the quasi-static fracture toughness of PLA decreased while its crystallinity increased. It was confirmed by the authors that the quenched PLA sample with *X*_c_ = 2.7% could generate extensive multiple crazes in the crack-tip region under quasi-static loading. However, with the increase of *X*_c_, the formation of multiple crazes in the crack-tip region was drastically suppressed. Moreover, the crack growth mainly directly propagated through the spherulites which formed firmly with each other, resulting in a decrease of the fracture toughness by increasing the crystallinity. In this paper, for the annealed PLA/TPEE (80/20) samples, it could be supposed that the high *X*_c_ and poor interfacial adhesion further sped up crack growth and greatly weakened the tensile ductility of the annealed PLA/TPEE samples with high crystallinity.

As seen in Figure 7c, while the *X*_c_ increased from 5% to 36%, the elongation at break of the PLA/TPEE/ADR (80/20/0.5) sample decreased from 250% to 146%. Compared with the annealed PLA/TPEE (80/20) samples with the maximum *X*_c_ (40%), the annealed PLA/TPEE/ADR (80/20/0.5) samples with the maxiumu *X*_c_ (36%) showed a higher elongation at break that increased by 421%. It was clear that the increased brittleness resulting from the increased *X*_c_ weakened the ductility of the PLA/TPEE/ADR (80/20/0.5) samples. However, similar to the quenched PLA/TPEE/ADR (80/20/0.5) sample (Figure 6b), the annealed PLA/TPEE/ADR (80/20/0.5) samples also presented a marked improvement in interfacial adhesion and a significant reduction in TPEE domain size, as shown in Figure 9b. Therefore, compared to the annealed PLA/TPEE (80/20) samples, the annealed PLA/TPEE/ADR (80/20/0.5) sample kept a higher tensile ductility due to the improved interfacial adhesion. First, the enhanced interfacial adhesion of the PLA matrix and TPEE domains resulted from the reactive compatibilization of ADR reduced the interface debonding which was a main form of interfacial crack growth of polymer blends. Second, the improved interfacial compatibility decreased the interfacial tension and reduced the TPEE domain size significantly. According to the crazing with shear yielding mechanism, when content of TPEE remained unchanged, the size reduction of the TPEE domain certainly led to a significant increase in the number of TPEE particles in PLA matrix, as indicated in Figure 9. This promoted the massive generation of crazes/shear bands, thereby allowing for a considerable development of plastic deformation [25,26]. Furthermore, the smaller TPEE particles and the improved interfacial adhesion also enhanced the yielding ability and shear plastic deformation of PLA matrix under tensile stress. Therefore, although the PLA/TPEE/ADR (80/20/0.5) samples with high *X*_c_ also exhibited a tendency to craze and prematurely fracture under the tensile stress, the improved interfacial adhesion and smaller TPEE domains still could effectively terminate the craze growth and restrain the nucleation and propagation of cracks to some extent, as well as enhance the yielding ability. As a result, the annealed PLA/TPEE/ADR (80/20/0.5) samples with high *X*_c_ showed a better tensile ductility than the annealed PLA/TPEE (80/20) sample without ADR. Moreover, while increasing ADR loading to 1.0 from 0.5 phr, the elongation at break of the annealed PLA/TPEE/ADR (80/20/1.0) sample remained almost unchanged.

As a result, the crystallized PLA/TPEE/ADR blends with excellent heat resistance and high tensile ductility were obtained by annealing and in situ reactive compatibilization.

## 4. Conclusions

The combined influences of crystallinity and reactive compatibilizer, ADR on the morphology, tensile performance, and heat resistance of the PLA/TPEE (80/20) blends were investigated. Annealing was performed to increase crystallinity of the samples. First, DSC measurement was performed to research the isothermal cold crystallization behavior and kinetics of the PLA/TPEE/ADR blends. Then, WAXD, SEM, tensile test, and VST measurements were carried out to research the influences of crystallinity and ADR loading on the morphology, tensile performance, and heat resistance of the PLA/TPEE blends. The differences in tensile fracture behaviors among the quenched and annealed PLA/TPEE samples with and without ADR were discussed in detail. The conclusions obtained are as follows:

(1) The crystallization kinetics results indicated that the addition of ADR decreased the cold crystallization rate of the samples because the chain extension and branching effects of ADR reduced the mobility and arrangement ability of PLA macromolecule segments and chains. For example, *t*_1/2_ of the PLA/TPEE (80/20) sample without ADR was 6.9 min. However, while increasing ADR loading up to 1 phr, *t*_1/2_ of the PLA/TPEE/ADR (80/20/1.0) sample significantly increased up to 11.2 min. In addition, it was confirmed that the increase in crystallization temperature could accelerate the cold crystallization of the PLA/TPEE samples with and without ADR.
(2) With the increasing of ADR loading from 0 up to 1 phr, the maximum crystallinity of the annealed PLA/TPEE/ADR samples also decreased from 40% to 34%. For the PLA/TPEE sample, its heat resistance enhanced greatly while the crystallinity increased from 4% to 40%. Meanwhile, the tensile ductility of the crystallized PLA/TPEE sample became very poor (ε = 28%) due to the embrittlement with increased crystallinity and the incompatibility between PLA and TPEE. As a comparison, the annealed PLA/TPEE/ADR samples with high crystallinity kept a higher tensile ductility because ADR improved the interfacial compatibility and refined the TPEE domains. For example, while the crystallinity increased from 5% up to the maximum value of 36%, the VST value of the PLA/TPEE/ADR (80/20/0.5) sample markedly increased from 58 to 136 °C. Meanwhile, the highly crystallized sample still presented a higher tensile ductility with an elongation at break of 146%. Moreover, the chain extension effect of ADR and increased crystallinity also presented a synergistic enhancement on the tensile strength of the PLA/TPEE samples.

## Figures and Tables

**Table 1:** Data of isothermal cold crystallization kinetics for PLA/TPEE/ADR blends.

Sample	*T*_c_ (°C)	Crystallization induction time (*t*_induce_, min)	*n*	*k* × 10^3^ (min^−1^)	*t*_1/2_ (min)
PLA/TPEE (80/20)	80	8.7	2.5	5.9	6.9
	82	4.1	2.4	17.8	4.7
	84	2.8	2.3	39.9	3.4
	86	1.9	2.2	84.0	2.6
PLA/TPEE/ADR (80/20/0.5)	80	10.3	2.5	2.0	10.1
	82	6.5	2.5	4.9	7.0
	84	4.8	2.5	13.1	5.0
	86	2.7	2.3	26.3	4.0
PLA/TPEE/ADR (80/20/1.0)	80	11.5	2.3	2.7	11.2
	82	9.0	2.3	4.0	9.4
	84	5.5	2.4	6.1	7.0
	86	3.3	2.5	9.7	5.6
